# Characteristics of healthcare workers and health facilities associated with inaccurate recording of malaria rapid diagnostic test results: a multi-country study

**DOI:** 10.1186/s12936-025-05674-2

**Published:** 2025-11-27

**Authors:** Sunday Atobatele, Arthur Mpimbaza, Corine Ngufor, William Yavo, Abibatou Konate-Toure, Idelphonse Ahogni, Nelson Ssewante, Evelyn Orya, Ese Akpiroroh, Onyebuchi Okoro, Bosco Agaba, Augustin Kpemasse, Jacques Agnon, Antoine Mea Tanoh, Cyriaque Affoukou, Jimmy Opigo, Godwin Ntadom, Hilary Okagbue, Eugene C. Eugene, John J. Aponte, Emily Hilton, Natalie Galles, Radina Soebiyanto, Shawna Cooper, Chukwu Okoronkwo, Michael Humes, Kevin Griffith, Kim A. Lindblade, Sidney Sampson

**Affiliations:** 1 Sydani Group, Abuja, Nigeria; 2 Sydani Institute for Research and Innovation, Abuja, Nigeria; 3 Child Health and Development Centre, College of Health Sciences, Makerere University, Kampala, Uganda; 4 Centre de Recherche Entomologique de Cotonou, Cotonou, Benin; 5 Institut National de Santé Publique, Abidjan, Côte d’Ivoire; 6 National Malaria Elimination Programme, Abuja, Nigeria; 7 National Malaria Control Division, Kampala, Uganda; 8 Programme National de Lutte Contre Le Paludisme, Cotonou, Benin; 9 Programme National de Lutte Contre Le Paludisme, Abidjan, Côte d’Ivoire; 10 PATH, Geneva, Switzerland; 11 PATH, Seattle, WA USA; 12 U. S. President’s Malaria Initiative, USAID, Washington, DC, USA; 13 Audere, Seattle, WA USA; 14 Department of Medical Laboratory Science, Faculty of Medicine, Mbarara University of Science and Technology, Mbarara, Uganda

## Abstract

**Background:**

Malaria rapid diagnostic tests (RDTs) have improved case management and surveillance across sub-Saharan Africa by reducing presumptive treatment and enhancing diagnostic specificity. However, healthcare workers’ (HCWs) concerns about limitations of RDTs, the lack of other diagnostic tools and patient expectations may result in non-adherence to RDT outcomes in treatment decisions. This study aimed to determine associations between HCW characteristics and the accuracy of recording malaria RDT results.

**Methods:**

A multi-country, mixed-methods observational study was conducted in 64 public health facilities across Benin, Côte d’Ivoire, Nigeria, and Uganda between June and December 2023. HCW demographic characteristics, attitudes and perceptions of RDTs, and proficiency performing RDTs were collected via surveys and structured observations. Completed RDTs were photographed, interpreted by a trained panel, and compared with health facility registers. Multivariable logistic regression models were used to identify factors associated with misrecording.

**Results:**

Among more than 100,000 RDTs performed by 499 HCWs, 5.1–7.3% of results were misrecorded as positive, and 0.7–3.7% were misrecorded as negative. The test positivity rate (TPR) was highest in Côte d’Ivoire (59.7%) and lowest in Nigeria (45.3%). Overall agreement on RDT results between the external panel and the result recorded by HCWs in the health facility register ranged from 90.2% in Nigeria to 94.3% in Benin. Misrecording of negative or invalid results as positive varied by country. In Benin, older HCWs and those with stronger malaria knowledge were less likely to misrecord, but HCWs who believed patients should still be treated after a negative test were more likely to do so. In Côte d’Ivoire, community health workers had higher odds of misrecording, while higher education reduced the risk. In Nigeria, medical auxiliary staff were less likely to misrecord than other cadres. In Uganda, misrecording was more common in high-volume, high-positivity facilities and among HCWs recently observed by a supervisor.

**Conclusion:**

Misrecording of RDT results is influenced by a combination of individual, contextual, and systemic factors, with differing patterns for results misrecorded as positive and negative. Improving malaria surveillance will require interventions that address both HCW behaviour and broader facility- and system-level influences.

**Supplementary Information:**

The online version contains supplementary material available at 10.1186/s12936-025-05674-2.

## Background

Malaria rapid diagnostic tests (RDTs) have significantly improved malaria case management and surveillance in sub-Saharan Africa over the past 15 years by reducing presumptive treatment and enhancing diagnostic specificity [1, 2]. However, RDT limitations, such as their inability to detect very low-density infections or parasite strains lacking genes for target antigens, have undermined the confidence of healthcare workers (HCWs) in the reliability of their results, leading many to disregard RDT outcomes when determining patient treatment [3–6]. Patient care may suffer when HCWs do not adhere to RDT results, leading to unnecessary antimalarial use and posing challenges in settings with limited stock. Additionally, surveillance quality may be sub-optimal if data recorded in health facility registers on patient management are not consistent with diagnostic outcomes.

HCWs exhibit varying levels of adherence with RDT results when making treatment decisions. Studies conducted in sub-Saharan Africa revealed that the rate at which healthcare workers disregarded negative RDT results and inappropriately prescribed antimalarial medications ranged widely from as low as 0.1% to as high as 81% [7–9]. This suggests significant inconsistencies in adherence to diagnostic guidelines, which may be influenced by several healthcare workers, patients, and systemic factors [3, 10, 11].

Healthcare providers may prescribe antimalarials after a negative RDT if they feel they have no other treatment options or if they retain doubts about the accuracy of RDTs [9, 12]. Pressures and perceived expectations from patients, caregivers, or communities also influence HCWs' adherence to RDT results [7]. In addition, health system factors like commodity stockouts and the use of diagnostic alternatives to RDTs such as clinical diagnosis and microscopy, also affect providers’ interpretation and use of RDT results [5, 13].

There are some limitations to previous efforts to understand which factors are most strongly associated with non-adherence to RDT results for treatment and misrecording RDT results in health facility registers. Relying on record reviews is likely to underestimate the problem: if HCWs have misrecorded RDT results to align with the treatment they provided, record reviews can only capture instances where HCWs documented treatment that was not aligned to the RDT result. Another approach is the use of re-interview methods that include selecting patients that have exited a health facility and conducting a second RDT by the interviewer [14]. However, in this approach, the original RDT used by the HCW is not directly observed. This introduces possible errors in misclassification, as the results may differ from the initial test. Moreover, re-interview studies may have smaller sample sizes, limiting their generalizability [15]. In addition, the Hawthorne effect, whereby HCWs align their behaviour with what is expected due to the awareness of being observed, may further distort findings, reducing the reliability of the data [16]. These limitations highlight the need for more robust approaches to accurately assess and address the factors influencing non-adherence to, and misrecording of, RDT results.

To further investigate factors that influence the misrecording of RDT results, a multi-country evaluation of the accuracy of malaria RDT results was conducted in health facility registers in Benin, Côte d’Ivoire, Nigeria and Uganda. The study aimed to determine associations between HCW characteristics and the accuracy of recording malaria RDT results.

## Methods

### Study design

This was a multi-country, mixed-methods observational study conducted between June and December 2023. The study integrated quantitative data from HCWs, health facility registers, and health management information systems (HMIS) of each of the four countries studied, with qualitative insights where relevant. Details of the methods are presented elsewhere [17].

### Study setting

This study was conducted in eight regions across four sub-Saharan African countries: Benin (Borgou and Zou), Côte d'Ivoire (Kabadougou and Mé), Nigeria (Oyo and Sokoto), and Uganda (Busoga and Lango). To reflect the diversity of malaria transmission within each country, research teams, working in collaboration with the national malaria programmes and the U.S. President’s Malaria Initiative (PMI), purposively selected two states or regions per country, taking into account differences in malaria transmission. In Benin, Côte d’Ivoire and Nigeria, only areas of the country where malaria RDTs and antimalarial medicines were supported by PMI were eligible.

### Selection of health facilities and HCWs

Within each selected region, two districts were purposively chosen. Public health facilities in these districts were eligible if they had reported malaria data to the HMIS for at least 9 out of 12 months over each of the previous two years and had performed a minimum of 50 RDTs per month. Eligible health facilities were stratified into four groups based on outpatient volume and test positivity rate from the previous year. One health facility per stratum was randomly selected in each district, yielding a total of 16 health facilities per country.

HCWs in selected health facilities were considered eligible to participate if they reported current or likely future involvement in performing RDTs or recording their results.

### Study procedures

Data were collected by trained research assistants. Before the start of the study in each country, a baseline survey was undertaken to characterize the conditions and capacities of each health facility. A knowledge, attitudes, perceptions and behaviour (KAPB) survey was conducted with eligible HCWs to gather information on their demographic characteristics, professional qualifications, and beliefs and attitudes with respect to RDTs. To determine HCW proficiency with RDTs, a research assistant observed each HCW performing an RDT and rated their performance against a previously validated, 19-point checklist [18].

Over the study period, research assistants photographed each RDT performed in the health facility shortly after interpretation by a HCW, without disrupting patient care or interacting with patients. Each RDT was labelled on the back with a unique barcode; a matching label was affixed to the patient’s corresponding outpatient department (OPD) register entry to enable linkage.

RDT images were sent for independent review to a trained panel of interpreters. Panelists assessed each image and determined the result based on the presence or absence of control and test line(s). All panelists underwent training and passed a certification test. Quality control included full review of all images and re-review of a random 30% sample of images, with flagged cases returned for reassessment.

### Data collection instruments

The facility survey, KAPB survey and structured observation of RDT proficiency were implemented on smartphones using KoboToolbox (Kobo, Cambridge, MA USA) with several validation features, including range and logic checks. The KAPB questionnaire collected information on HCWs training and experience, knowledge of malaria transmission and case management, attitudes toward RDTs, perceptions of RDT accuracy, frequency of performing and recording RDTs, subjective norms regarding RDT use, training and supervision. A random, unique identification number was assigned to each HCW who completed the KAPB survey to link data across data collection instruments.

The collection of RDT images and associated data from the OPD register were completed using the HealthPulse application (Audere, Seattle, WA USA), a digital RDT reader customized for this study and installed on smartphones. The HealthPulse application has been validated in an earlier study [19]. Its core features include an artificial intelligence-powered (AI) algorithm for interpreting RDT results images and an image quality assurance (IQA) component that uses computer vision and machine learning to verify image quality (e.g., blurry images) and RDT type. The output of the AI algorithm was not shared with research assistants, HCWs, external panelists, or study investigators (with the exception of SC) during the study period and did not influence clinical decision-making. On-device IQA was used to prompt research assistants to retake poor-quality images.

After using the HealthPulse application to photograph RDTs, research assistants used it to scan the barcodes placed against patient records in the OPD register and enter anonymized patient data, including demographics, diagnosis, and treatment. The unique code for the HCW who recorded the RDT data in the health facility register was also recorded.

### Data management and analysis

The RDT result interpreted by the external panel was treated as the reference standard. If the panel classified a result as negative or invalid but the HCW recorded it as positive, the outcome was categorized as ‘misrecorded as positive’. Conversely, if the panel classified the result as positive or invalid but the HCW recorded it as negative, the outcome was categorized as ‘misrecorded as negative’. HCWs who recorded RDT results were considered primarily responsible for the accuracy of the documentation. Accordingly, only HCWs who documented at least one RDT result observed during the study were included in the analysis. Their characteristics were examined in relation to the likelihood of misrecording an RDT result.

The geographic coordinates of each study facility were used to calculate average 2022 *Plasmodium falciparum* parasite prevalence for children 2–10 years (PfPR_2-10_) within 5 km of the facility using the malariaAtlas package in R (R Foundation for Statistical Computing, Vienna, Austria) [20]. Facilities were then grouped into terciles based on the distribution of PfPR_2–10_ values across the 16 facilities within each country. PfPR_2-10_ ranged between 30 and 39% in Benin, 19% and 34% in Côte d’Ivoire, 16% and 35% in Nigeria, and 7% and 38% in Uganda. Continuous variables, including age, years of experience, and hours worked per week, were categorized into standard predefined groups.

A 6-point malaria knowledge score was created out of responses to open-ended questions about the causes of malaria, preventive measures, and signs and symptoms of uncomplicated and severe malaria. A score of three or fewer was considered low knowledge. The 19-point RDT proficiency score was divided into terciles based on the distribution of scores in each country. Questions of perceptions or beliefs based on a five-point Likert scale were collapsed into three categories: ‘agree or strongly agree’, ‘neutral’ or ‘disagree or strongly disagree’.

As the proportion of results misrecorded as negative was substantially lower than those misrecorded as positive across all four countries [17], it was hypothesized that the factors associated with each type of error would differ. Therefore, analyses were conducted separately using two datasets: one was restricted to records where the panel classified the outcome as negative or invalid (to examine factors associated with misrecording as positive), and one was restricted to results classified as positive or invalid (to examine factors associated with misrecording as negative).

Logistic regression was used to model the odds of misrecording an RDT result. Nineteen independent variables included HCW characteristics such as sex, age, experience, knowledge, attitudes, perceptions and behaviours were examined (Table 1**)**. To account for clustering of RDT results by HCW, standard errors and 95% confidence intervals (CI) were adjusted using cluster-robust variance estimators (via the vcovCL function) in R.

**Table 1:** Descriptive characteristics of healthcare workers and their knowledge, attitudes, perceptions and behaviours

Variable	Benin N = 182 n (%)	Côte d’ Ivoire N = 110 n (%)	Nigeria N = 114 n (%)	Uganda N = 93 n (%)
*HCW characteristics*				
Sex				
Female	128 (70.3)	71 (64.5)	86 (75.4)	46 (49.5)
Male	54 (29.7)	39 (35.5)	28 (24.6)	47 (50.5)
Age (years)				
< 30	65 (35.7)	41 (37.3)	54 (47.4)	22 (23.7)
30–39	58 (31.9)	54 (49.1)	35 (30.7)	36 (38.7)
40–49	46 (25.3)	11 (10.0)	17 (14.9)	26 (28.0)
50–59	13 (7.1)	4 (3.6)	8 (7.0)	9 (9.7)
60 +	0 (0.0)	0 (0.0)	0 (0.0)	0 (0.0)
Occupational category				
Medical doctor	7 (3.8)	0 (0.0)	0 (0.0)	0 (0.0)
Clinical officer	0 (0.0)	0 (0.0)	0 (0.0)	9 (9.7)
Nurse	42 (23.1)	33 (30.0)	11 (9.6)	49 (52.7)
Midwife	26 (14.3)	17 (15.5)	0 (0.0)	0 (0.0)
Medical auxiliary staff	81 (44.5)	24 (21.8)	10 (8.8)	10 (10.8)
Lab technician or assistant	0 (0.0)	0 (0.0)	28 (24.6)	5 (5.4)
Community health worker	2 (1.1)	4 (3.6)	38 (33.3)	8 (8.6)
Non-medical staff^a^	24 (13.2)	32 (29.1)	27 (23.7)	12 (12.9)
Highest educational level achieved				
Primary school or below	123 (67.6)	5 (4.5)	0 (0.0)	3 (3.2)
Secondary school	39 (21.4)	39 (35.5)	28 (24.6)	14 (15.1)
University	20 (11.0)	66 (60.0)	86 (75.4)	76 (81.7)
Experience (years)				
0–1	24 (13.2)	49 (44.5)	27 (23.7)	8 (8.6)
2–4	49 (26.9)	31 (28.2)	25 (21.9)	12 (12.9)
5–9	32 (17.6)	20 (18.2)	26 (22.8)	25 (26.9)
10 +	77 (42.3)	10 (9.1)	36 (31.6)	48 (51.6)
Amount worked in a week (hours)				
0–19	4 (2.2)	0 (0.0)	4 (3.5)	7 (7.5)
20–39	3 (1.6)	14 (12.7)	26 (22.8)	11 (11.8)
40–49	24 (13.2)	25 (22.7)	54 (47.4)	49 (52.7)
50 +	151 (83.0)	71 (64.5)	30 (26.3)	26 (28.0)
*Knowledge and proficiency*				
Knowledge index				
Lowest	92 (50.5)	74 (67.3)	65 (57.0)	55 (59.1)
Highest	90 (49.5)	36 (32.7)	49 (43.0)	38 (40.9)
RDT proficiency (tercile)				
Lowest	60 (33.5)	37 (33.9)	35 (34.0)	29 (33.3)
Middle	60 (33.5)	36 (33.0)	34 (33.0)	29 (33.3)
Highest	59 (33.0)	36 (33.0)	34 (33.0)	29 (33.3)
*Attitudes and beliefs*				
Is it possible for a patient to have a negative RDT test when they actually have a malaria infection?				
Yes	162 (91.0)	79 (80.6)	64 (56.6)	74 (79.6)
No	16 (9.0)	19 (19.4)	49 (43.4)	19 (20.4)
Do you think you should treat a patient with an antimalarial even if their RDT returns a negative result?				
Yes	32 (17.8)	37 (34.9)	23 (21.1)	11 (11.8)
No	148 (82.2)	69 (65.1)	86 (78.9)	82 (88.2)
I have enough time to use malaria RDTs correctly in this facility for all patients who need them				
Agree or strongly agree	161 (88.5)	87 (79.1)	100 (87.7)	81 (87.1)
Neutral	16 (8.8)	15 (13.6)	4 (3.5)	9 (9.7)
Disagree or strongly disagree	5 (2.7)	8 (7.3)	10 (8.8)	3 (3.2)
If an RDT is negative, I have medicines at this facility that I can use to treat the patient other than antimalarials				
Agree or strongly agree	135 (74.2)	93 (84.5)	95 (83.3)	76 (81.7)
Neutral	21 (11.5)	7 (6.4)	3 (2.6)	13 (14.0)
Disagree or strongly disagree	26 (14.3)	10 (9.1)	16 (14.0)	4 (4.3)
*Subjective norms*				
I use malaria RDTs because the patients at this health facility expect me to use them				
Agree or strongly agree	43 (23.6)	17 (15.5)	55 (48.2)	29 (31.2)
Neutral	16 (8.8)	14 (12.7)	7 (6.1)	16 (17.2)
Disagree or strongly disagree	123 (67.6)	79 (71.8)	52 (45.6)	48 (51.6)
I use malaria RDTs because my supervisor expects me to use them				
Agree or strongly agree	44 (24.2)	28 (25.5)	49 (43.0)	38 (40.9)
Neutral	17 (9.3)	11 (10.0)	7 (6.1)	14 (15.1)
Disagree or strongly disagree	121 (66.5)	71 (64.5)	58 (50.9)	41 (44.1)
I use malaria RDTs because the national malaria treatment guidelines require me to use them				
Agree or strongly agree	111 (61.0)	60 (54.5)	93 (81.6)	76 (81.7)
Neutral	24 (13.2)	14 (12.7)	0 (0.0)	9 (9.7)
Disagree or strongly disagree	47 (25.8)	36 (32.7)	21 (18.4)	8 (8.6)
*Behaviours*				
Frequency of performing RDTs				
Very often (every day)	163 (91.1)	59 (55.7)	96 (84.2)	50 (58.8)
Once in a while to often	16 (8.9)	47 (44.3)	18 (15.8)	35 (41.2)
Never	3 (1.6)	4 (3.6)	0 (0.0)	8 (8.6)
Frequency of recording RDT results				
Very often (every day)	154 (91.7)	60 (85.7)	70 (84.3)	58 (67.4)
Once in a while to often	14 (8.3)	10 (14.3)	13 (15.7)	28 (32.6)
Never	14 (7.7)	40 (36.4)	31 (27.2)	7 (7.5)
*Training and supervision*				
Received RDT training in the past year				
Yes	84 (46.2)	68 (61.8)	89 (78.1)	39 (41.9)
No	98 (53.8)	42 (38.2)	25 (21.9)	54 (58.1)
Supervisor observed their performance of an RDT in the past year				
Yes	67 (36.8)	31 (28.2)	74 (64.9)	30 (32.3)
No	115 (63.2)	79 (71.8)	40 (35.1)	63 (67.7)

RDT: Rapid Diagnostic Test

^a^The non-medical staff category includes students, interns and volunteers

A subset of HCW characteristics was selected for inclusion in the final model selection process based on evidence of a statistical association in univariate analysis with at least one country. Several variables were excluded due to collinearity or convergence issues (Supplemental Table 1). Two facility-level factors, region and stratum, were forced into all models to account for the sampling design. An additional facility-level variable, PfPR2-10, and two patient-level variables, sex and age, were also considered for inclusion. Final model selection was performed using Akaike-information criterion (AIC)-based stepwise logistic regression with the stepAIC function in the MASS package in R to identify key predictors associated with misrecording of positive and negative results in each country [21]. The stepAIC procedure optimizes model selection by minimizing AIC, a measure that balances model fit and complexity. AIC penalizes inclusion of additional variables that do not substantially improve model fit. Adjusted odds ratios (ORs) from the final model were visualized in dot plots to facilitate comparison across countries and between the two directions of misrecording.

### Ethical considerations

Written, informed consent was obtained from participating HCWs who were informed that anonymous identifiers would be used to link information from the KAPB survey and RDT observation to RDTs they recorded. No patients were consented by the study team as the RDT images and data from the health facility registers were recorded anonymously and obtained from secondary data sources, respectively. No personally identifiable information was obtained from patient records.

The PATH institutional review board approved the multi-country study protocol. In Benin, the Comité National d’Ethique pour la Recherche en Santé provided approval. In Côte d’Ivoire, the Comité National d'Éthique des Sciences de la Vie et de la Santé approved the study. In Nigeria, approval was received from Oyo State Ministry of Health Research Ethics Committee, Sokoto State Health Research Ethics Committee, and the National Health Research Ethics Committee of Nigeria. The Uganda National Council for Science and Technology and Vector Control Division-Research & Ethics Committee approved the study in Uganda.

## Results

### HCW characteristics

In total, 499 HCWs recorded at least one RDT observed in the study across 64 health facilities in Benin, Côte d'Ivoire, Nigeria, and Uganda. The majority of HCWs were females, except in Uganda where there was a fairly equal distribution of men and women (Table 1). In Uganda, 23.7% of HCWs were less than 30 years old, compared to 35.7% in Benin, 37.3% in Côte d’Ivoire and 47.4% in Nigeria. None of the HCWs across the four countries was 60 years or older. The occupational cadre of HCWs varied by country: the most common cadre in Benin were medical auxiliary staff (44.5%) and nurses (23.1%); in Côte d’Ivoire, nurses (30.0%) and nonmedical staff (29.1%); in Nigeria, community health workers (33.3%) and lab technicians or assistants (24.6%); and in Uganda, nurses (52.7%) and nonmedical staff (12.9%). Only Benin reported medical doctors (3.8%) among the HCWs. Most HCWs had a tertiary level of education although HCWs in Benin (67.6%) mostly had a primary level of education.

The majority of respondents in all countries were in the lowest category of the knowledge index (Table 1). Most HCWs (range: 56.6–91.0%) believed that it is possible for a patient to have a negative RDT test when they actually have a malaria infection, but a much lower proportion (range: 11.8–34.9%) believed that such patients should be treated with an antimalarial medicine. More than 79% of the respondents in each country agreed or strongly agreed that they have sufficient time to perform malaria RDTs, and a similarly high proportion (range: 74.2–84.5%) felt they had other medicines in the facility besides antimalarials that they could use to treat their patients.

More HCWs agreed or strongly agreed that motivation to use RDTs was derived from national malaria treatment guidelines (range: 54.5–81.7%) than patients (range: 15.5–48.2%) or their supervisors (range: 24.2–43.0%) (Table 1).

There were differences between countries with respect to the frequency that HCWs performed RDTs. In Benin and Nigeria, almost all (range: 84.2–91.1%) performed RDTs very often whereas this proportion was lower in Côte d’Ivoire (55.7%) and Uganda (58.8%) (Table 1). However, a large proportion of HCWs across all four countries recorded RDT results very often (range: 67.4–91.7%).

Fewer than half of respondents in Benin (46.2%) and Uganda (41.9%) reported receiving RDT training in the preceding year, compared to 61.8% in Côte d’Ivoire and 78.1% in Nigeria (Table 1). In Nigeria, over 60% of HCWs reported that a supervisor had observed their performance of an RDT in the past year, whereas this figure was below 37% in the other three countries.

### Agreement on RDT results

More than 100,000 RDTs were observed over the course of the study (Table 2). The number of RDTs performed during the study varied between 11,161 in Côte d’Ivoire and 37,137 in Uganda. The test positivity rate was highest in Côte d’Ivoire (59.7%) and Uganda (56.3%) and lowest in Nigeria (45.3%). Very few results (between 0 and 0.4% per country) were classified as invalid by the external panel. Overall agreement on RDT results between the external panel and the result recorded by HCWs in the health facility register ranged from 90.2% in Nigeria to 94.3% in Benin. Between 5.1% of results in Benin and 7.3% of results in Uganda were misrecorded as positive, while between 0.7% RDT results in Benin and 3.7% in Nigeria were misrecorded as negative.

**Table 2:** Numbers of RDTs observed and comparison of outcomes between the external panel and HCWs, 2023

	Benin	Cote d’ Ivoire	Nigeria	Uganda
Number of RDTs observed during the study	35,720	11,161	18,319	37,137
Positive results (external panel) (%)	17,913 (50.1)	6662 (59.7)	8302 (45.3)	20,913 (56.3)
Negative results (external panel) (%)	17,793 (49.8)	4467 (40.0)	9953 (54.3)	16,107 (43.3)
Invalid results (external panel) (%)	141 (0.0)	32 (0.3)	64 (0.4)	117 (0.3)
RDT results in agreement between external panel and HCWs (%)	33,671 (94.3)	10,281 (92.1)	16,515 (90.2)	33,743 (90.9)
True negative/invalid results misrecorded as positive (%)	1805 (5.1)	662 (5.9)	1129 (6.2)	2698 (7.3)
True positive/invalid results misrecorded as negative (%)	244 (0.7)	218 (2.0)	675 (3.7)	696 (1.9)

HCWs: healthcare workers; RDTs: Rapid diagnostic tests

### Analysis of factors associated with misrecording negative or invalid RDT results as positive

#### Univariate

Region was statistically associated with results misrecorded as positive in Benin, Nigeria and Uganda, but not in Côte d’Ivoire (Table 3). In Benin, HCWs in health facilities located in strata with both high patient volume and high test positivity had higher odds of misrecording RDT results as positive compared to those in the other three strata. A similar trend was observed in Uganda, where HCWs in high-volume, high-positivity strata had significantly higher odds of misrecording results as positive compared to HCWs in strata with low positivity, though not when compared to other high-positivity strata. Higher PfPR_2–10_ in the area surrounding the facility was associated with increased odds of misrecording results as positive in Benin, Côte d’Ivoire and Uganda, although not all confidence intervals excluded the null result. In contrast, in Nigeria, increasing PfPR_2–10_ was associated with decreased odds of misrecording a true negative result as positive, although all results included the null.

**Table 3:** Factors associated with misrecording negative or invalid RDT results as positive*

Variable	Benin OR (95% CI)	Côte d’ Ivoire OR (95% CI)	Nigeria OR (95% CI)	Uganda OR (95% CI)
*Facility characteristics*				
Region				
Zou (BEN), Me (CIV), Oyo (NGA), Busoga (UGA)	**0.5 (0.3, 0.7)**	1.4 (0.8, 2.5)	**0.5 (0.4, 0.8)**	**0.5 (0.4, 0.7)**
Borgou (BEN), Kabadougou (CIV), Sokoto (NGA), Lango (UGA)	1.0	1.0	1.0	1.0
Stratum				
High volume/High positivity rate	1.0	1.0	1.0	1.0
High volume/Low positivity rate	**0.2 (0.2, 0.4)**	1.1 (0.6, 2.0)	1.1 (0.5, 2.1)	**0.7 (0.5, 0.9)**
Low volume/High positivity rate	**0.5 (0.3, 0.8)**	1.9 (0.8, 4.4)	1.2 (0.6, 2.4)	1.0 (0.7, 1.4)
Low volume/Low positivity rate	**0.6 (0.3, 0.9)**	**2.5 (1.1, 5.6)**	1.0 (0.5, 2.1)	**0.7 (0.5, 0.9)**
Parasite prevalence (PfPR2-10) (tercile)				
Lowest	1.0	1.0	1.0	1.0
Middle	**2.1 (1.3, 3.2)**	1.4 (0.7, 2.6)	0.6 (0.4, 1.0)	**1.6 (1.1, 2.2)**
Highest	1.9 (1.0, 3.6)	1.6 (0.8, 3.3)	0.6 (0.4, 1.0)	**1.7 (1.2, 2.6)**
*HCW characteristics*				
Sex				
Female	0.6 (0.4, 1.0)	1.1 (0.6, 1.8)	**0.6 (0.4, 0.9)**	0.9 (0.7, 1.3)
Male	1.0	1.0	1.0	1.0
Age (years)				
< 30	1.0	1.0	1.0	1.0
30–39	0.8 (0.5, 1.2)	1.7 (1.0, 2.9)	1.2 (0.7, 2.0)	1.2 (0.8, 1.7)
40–49	**0.3 (0.2, 0.4)**	2.1 (1.0, 4.7)	0.9 (0.5, 1.7)	1.1 (0.7, 1.8)
50–59	**0.2 (0.1, 0.3)**	0.8 (0.2, 2.5)	0.8 (0.4, 1.8)	1.6 (0.9, 2.6)
Occupational category				
Medical doctor	0.6 (0.4, 1.1)	NA	NA	NA
Clinical officer	NA	NA	NA	1.1 (0.6, 1.9)
Nurse	**0.4 (0.2, 0.8)**	0.8 (0.4, 1.8)	0.5 (0.2, 1.3)	1.1 (0.7, 1.8)
Midwife	0.7 (0.3, 1.5)	1.7 (0.6, 5.0)	NA	NA
Medical auxiliary staff	**0.5 (0.3, 0.9)**	0.7 (0.3, 1.6)	**0.3 (0.1, 0.8)**	0.9 (0.4, 1.7)
Lab technician or assistant	NA	NA	0.7 (0.4, 1.2)	1.9 (0.8, 4.4)
Community health worker	0.7 (0.3, 1.7)	**2.1 (1.1, 4.1)**	0.9 (0.5, 1.6)	1.1 (0.7, 1.9)
Non-medical staff ^a^	1.0	1.0	1.0	1.0
Highest educational level achieved				
Primary school or below	1.0	1.0	NA	1.0
Secondary school	1.2 (0.6, 2.2)	**0.4 (0.3, 0.6)**	NA	**0.5 (0.3, 0.8)**
University	1.2 (0.8, 1.9)	**0.5 (0.3, 0.8)**	1.2 (0.8, 1.9)	0.9 (0.7, 1.1)
Experience (years)				
0–1	1.0	1.0	1.0	1.0
2–4	0.8 (0.4, 1.6)	1.0 (0.5, 2.2)	1.1 (0.5, 2.4)	0.9 (0.5, 1.9)
5–9	0.6 (0.3, 1.3)	2.0 (1.0, 3.9)	1.6 (1.0, 2.8)	1.1 (0.6, 1.8)
10+	**0.3 (0.2, 0.6)**	1.0 (0.5, 1.9)	1.2 (0.7, 2.1)	1.2 (0.7, 2.0)
Amount worked per week (hours)				
0–19	1.0	NA	1.0	1.0
20–39	0.8 (0.4, 1.7)	1.0	0.6 (0.4, 1.0)	0.7 (0.4, 1.2)
40–49	0.7 (0.4, 1.4)	1.9 (0.9, 4.4)	0.7 (0.5, 1.1)	0.8 (0.4, 1.4)
50+	1.0 (0.6, 1.7)	1.8 (0.9, 3.7)	0.7 (0.4, 1.2)	0.8 (0.5, 1.4)
*Knowledge and proficiency*				
Knowledge index				
Lowest	1.0	1.0	1.0	1.0
Highest	**0.5 (0.3, 0.8)**	**2.5 (1.3, 4.5)**	0.8 (0.5, 1.2)	1.0 (0.7, 1.4)
RDT proficiency (tercile)				
Lowest	1.0	1.0	1.0	1.0
Middle	1.2 (0.7, 2.1)	2.1 (1.0, 4.3)	0.8 (0.5, 1.2)	1.1 (0.8, 1.6)
Highest	0.9 (0.4, 1.7)	1.5 (0.8, 2.8)	0.9 (0.5, 1.5)	0.8 (0.5, 1.1)
*Attitudes and beliefs*				
Is it possible for a patient to have a negative RDT test when they actually have a malaria infection?				
Yes	0.8 (0.4, 1.5)	1.8 (1.0, 3.3)	1.0 (0.6, 1.6)	0.7 (0.6, 1.0)
No	1.0	1.0	1.0	1.0
Do you think you should treat a patient with an antimalarial even if their RDT returns a negative result?				
Yes	**2.1 (1.1, 4.0)**	1.2 (0.7, 2.1)	1.2 (0.7, 2.0)	0.7 (0.4, 1.2)
No	1.0	1.0	1.0	1.0
I have enough time to use malaria RDTs correctly in this facility for all patients who need them				
Agree or strongly agree	0.5 (0.2, 1.1)	1.1 (0.5, 2.1)	0.5 (0.2, 1.1)	**1.5 (1.1, 1.9)**
Neutral	**0.3 (0.1, 0.9)**	3.5 (1.0, 12.1)	**0.3 (0.1, 0.8)**	**1.7 (1.2, 2.6)**
Disagree or strongly disagree	1.0	1.0	1.0	1.0
If an RDT is negative, I have medicines at this facility that I can use to treat the patient other than antimalarials				
Agree or strongly agree	**2.2 (1.5, 3.4)**	0.8 (0.4, 1.7)	0.9 (0.4, 2.0)	1.0 (0.6, 1.5)
Neutral	2.0 (1.0, 3.7)	1.2 (0.5, 3.3)	1.2 (0.6, 2.5)	1.3 (0.7, 2.5)
Disagree or strongly disagree	1.0	1.0	1.0	1.0
*Subjective norms*				
I use malaria RDTs because the patients at this health facility expect me to use them				
Agree or strongly agree	1.0 (0.6, 1.7)	0.6 (0.3, 1.3)	1.3 (0.8, 2.2)	0.9 (0.7, 1.1)
Neutral	1.0 (0.5, 2.0)	2.4 (0.9, 6.6)	0.8 (0.4, 1.4)	**0.5 (0.3, 0.7)**
Disagree or strongly disagree	1.0	1.0	1.0	1.0
I use malaria RDTs because my supervisor expects me to use them				
Agree or strongly agree	0.8 (0.5, 1.2)	0.9 (0.4, 2.0)	1.3 (0.7, 2.1)	0.8 (0.6, 1.2)
Neutral	0.6 (0.3, 1.3)	**3.2 (1.5, 7.0)**	0.7 (0.4, 1.5)	1.1 (0.7, 1.7)
Disagree or strongly disagree	1.0	1.0	1.0	1.0
I use malaria RDTs because the national malaria treatment guidelines require me to use them				
Agree or strongly agree	0.9 (0.5, 1.6)	0.8 (0.5, 1.5)	0.6 (0.3, 1.2)	**0.6 (0.5, 0.8)**
Neutral	0.9 (0.4, 1.8)	**2.5 (1.2, 5.1)**	1.0	**0.4 (0.2, 0.8)**
Disagree or strongly disagree	1.0	1.0	NA	1.0
*Behaviours*				
Frequency of recording RDT results				
Very often (every day)	**0.5 (0.3, 0.8)**	1.3 (0.6, 2.6)	0.7 (0.4, 1.2)	1.2 (0.7, 1.9)
Once in a while to often	0.6 (0.3, 1.1)	**3.4 (1.4, 8.3)**	0.6 (0.2, 1.5)	0.8 (0.5, 1.4)
Never	1.0	1.0	1.0	1.0
*Training and supervision*				
Received RDT training in the past year				
Yes	1.1 (0.6, 1.8)	0.9 (0.5, 1.7)	0.8 (0.5, 1.3)	1.1 (0.8, 1.5)
No	1.0	1.0	1.0	1.0
Supervisor observed the performance of an RDT within the past year				
Yes	1.1 (0.6, 1.9)	1.2 (0.6, 2.2)	1.2 (0.8, 1.8)	**1.4 (1.1, 1.8)**
No	1.0	1.0	1.0	1.0
*RDT-level variables*				
Patient sex				
Female	0.9 (0.8, 1.1)	0.9 (0.8, 1.1)	1.0 (0.8, 1.1)	**0.8 (0.7, 0.8)**
Male	1.0	1.0	1.0	1.0
Patient age (years)				
< 5	1.0	1.0	1.0	1.0
5–14	**3.0 (2.4, 3.7)**	**2.2 (1.8, 2.9)**	**1.4 (1.1, 1.8)**	**1.6 (1.4, 1.9)**
15+	**1.9 (1.6, 2.4)**	1.0 (0.9, 1.2)	0.9 (0.7, 1.2)	**0.8 (0.7, 0.9)**

^*^Odds ratios and their 95% CI that exclude 1.0 are bolded

In Benin, older HCWs had reduced odds of misrecording results as positive but healthcare worker age was not associated with misrecording results as positive in any other country (Table 3). In both Benin and Nigeria, nurses had lower odds of misrecording results as positive in comparison to non-medical staff, while medical auxiliary staff in both Benin and Nigeria had lower odds of misrecording results as positive. In Côte d’Ivoire, community health workers were twice as likely as non-medical staff to misrecord results as positive. There was no consistent pattern by cadre observed in Uganda. Higher levels of education were associated with lower odds of misrecording positive results in Côte d’Ivoire and Uganda, although 95% CI for some estimates included the null. Neither years of experience nor the number of hours worked per week were associated with misrecording results as positive in the four countries, except that in Benin, the odds of misrecording declined as years of experience increased.

In Benin, HCWs with the highest level of knowledge of malaria had lower odds of misrecording results as positive, whereas in Côte d’Ivoire, the converse was true (Table 3). Proficiency in performing RDTs, as measured during structured observation, was not associated with the odds of misrecording RDT results as positive in any of the four countries.

There was no association between the belief that a patient with malaria could have a negative RDT result and the odds of misrecording a true negative result as positive in any country (Table 3). In Benin, HCWs who believed that patients should be treated with an antimalarial even if the RDT result was negative had significantly higher odds of misrecording results as positive but this finding was not replicated in the other countries. In Uganda, perceptions of having enough time to use malaria RDTs were associated with misrecording results as positive.

The expectations of patients and supervisors were not found to be associated with misrecording results as positive, although in Uganda, the subjective norms around national malaria treatment guidelines was associated with reduced odds of misrecording results as positive (Table 3). Training on RDTs within the past year was not associated with the odds of misrecording results as positive; however, in Uganda, HCWs whose supervisor observed the performance of an RDT within the last year were at increased odds of misrecording results as positive.

In Uganda, female patients were less likely to have their result misrecorded as positive (Table 3). The age of the patient on whom the RDT was performed was associated with the odds of misrecording results as positive across all countries. School-age children 5 to 14 years old were associated with 1.4 to 3.0 times the odds of misrecording RDT results as positive compared to children less than 5 years old. In Benin, patients 15 years and older were statistically associated with increased odds of misrecording results as positive but in Uganda, this age group was associated with decreased odds of misrecording results as positive compared to children less than 5 years old.

#### Multivariable

**Fig. 1:**
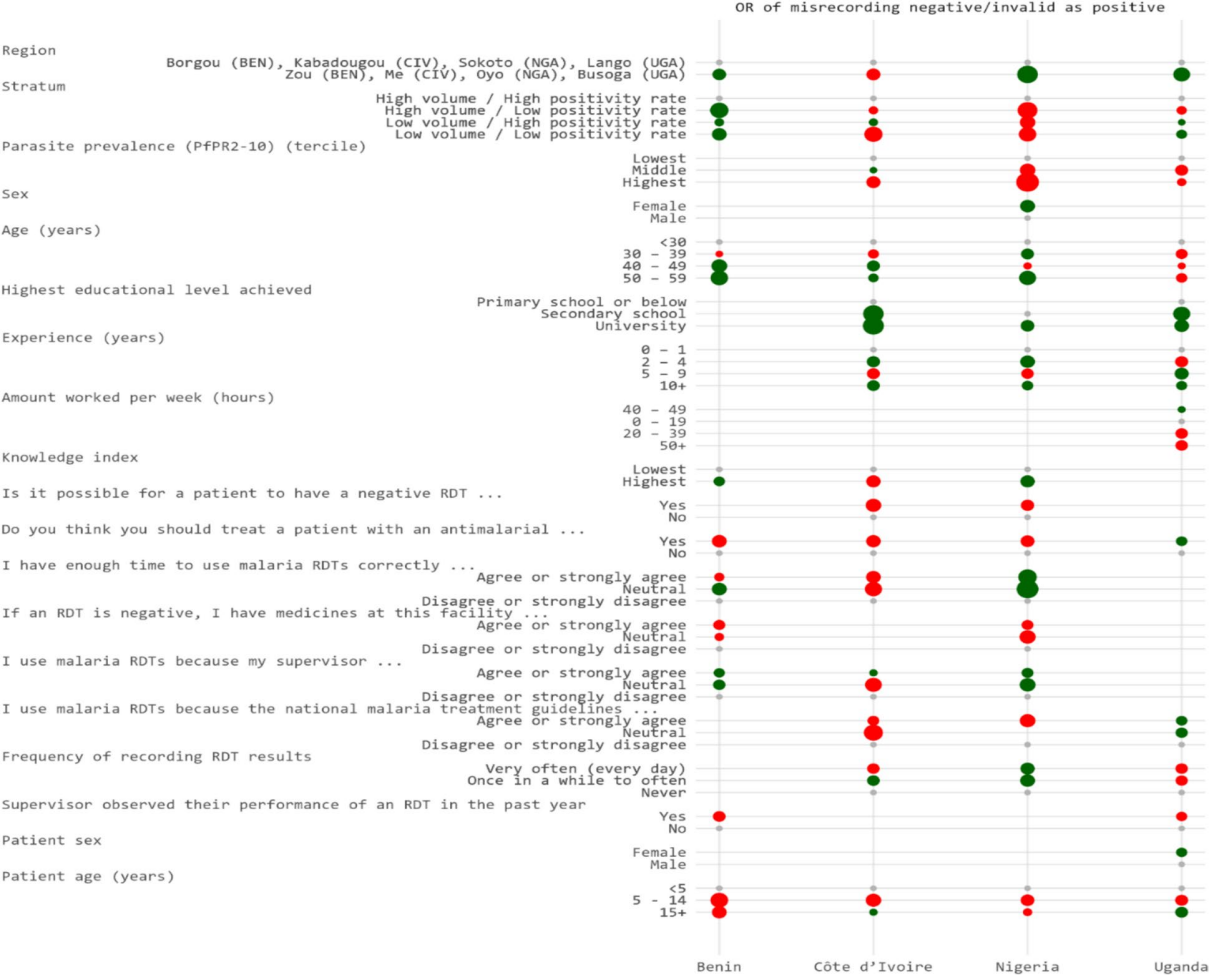
Dot plot of adjusted odds ratios for factors selected in final models of predictors of misrecording RDT results as positive by country, 2023

In multivariable models selected to balance model fit and complexity, three of the four countries retained PfPR_2-10_ as a predictor; the variable was not included in the model for Benin. The highest tercile of PfPR_2-10_ was associated with increased odds of misrecording a true positive RDT result as negative (Fig. 1).

Several HCW characteristics were retained in different country-specific models. However, only one HCW variable, age, was included in all four country models. Older HCWs in Benin and Côte d’Ivoire were less likely to misrecord results as positive, whereas the opposite was true in Uganda. In Nigeria, results varied by age category. For Côte d’Ivoire, Nigeria and Uganda, educational achievement was associated with reduced odds of misrecording RDT results as positive compared to those with only primary education or less (or secondary school in Nigeria).

With respect to knowledge, attitudes, beliefs and practices, one variable was retained in the model for all four countries. In Benin, Côte d’Ivoire and Nigeria, HCWs who agreed that patients should be treated with antimalarials even if their RDT result was negative were more likely to misrecord RDT results as positive, whereas in Uganda, they were less likely to misrecord results as positive. HCWs in Benin and Uganda who reported their supervisor observed their performance of an RDT in the past year were more likely to misrecord results as positive. Patient age remained a consistent factor: school age children (5–14 years old) in all countries, and patients aged 15 years and above in Benin and Nigeria, had higher odds of having their RDT result misrecorded as positive than children less than 5 years old.

### Analysis of factors associated with misrecording true positive RDT results as negative

#### Univariate

In comparison to the univariate analysis of factors associated with misrecording true negative results as positive, there were fewer factors consistently associated with misrecording true positive results as negative across the four countries (Table 4). Regional differences were important in Benin, Côte d’Ivoire and Nigeria but not in Uganda. Increasing PfPR_2-10_ was associated with reduced odds of misrecording results as negative in Côte d’Ivoire and Nigeria but some 95% confidence intervals included the null. Using malaria RDTs because the national malaria treatment guidelines require it was protective against misrecording results as negative in all countries but only statistically significant in Benin and Nigeria. HCWs who frequently recorded RDT results were more likely to misrecord results as negative in Nigeria and Uganda. Patient age was associated with the odds of misrecording results as negative: patients age 15 years and over were associated with OR of 1.3 to 3.0, although the 95% CI included the null in Nigeria. Conversely, patients age 5–14 had lower odds of misrecording results as negative in Benin and Nigeria.

**Table 4:** Factors associated with true positive or invalid RDT results misrecorded as negative

Variable	Benin OR (95% CI)	Côte d’ Ivoire OR (95% CI)	Nigeria OR (95% CI)	Uganda OR (95% CI)
*Facility characteristics*				
Region				
Zou (BEN), Me (CIV), Oyo (NGA), Busoga (UGA)	2.0 (1.2, 3.3)	0.5 (0.3, 0.9)	0.6 (0.4, 0.9)	1.0 (0.7, 1.3)
Borgou (BEN), Kabadougou (CIV), Sokoto (NGA), Lango (UGA)	1.0	1.0	1.0	1.0
Stratum				
High volume/High positivity rate	1.0	1.0	1.0	1.0
High volume/Low positivity rate	1.4 (0.9, 2.1)	0.9 (0.4, 1.9)	2.1 (1.2, 3.7)	0.7 (0.5, 1.0)
Low volume/High positivity rate	1.1 (0.5, 2.6)	0.8 (0.2, 2.9)	1.6 (0.9, 3.0)	0.7 (0.5, 1.2)
Low volume/Low positivity rate	0.5 (0.3, 0.9)	1.5 (0.4, 5.6)	2.4 (1.3, 4.6)	0.9 (0.6, 1.3)
Parasite prevalence (PfPR2-10) (tercile)				
Lowest	1.0	1.0	1.0	1.0
Middle	0.6 (0.4, 1.0)	0.3 (0.2, 0.6)	0.6 (0.3, 1.1)	1.1 (0.7, 1.5)
Highest	1.3 (0.8, 2.3)	0.3 (0.1, 0.7)	0.5 (0.3, 0.9)	1.1 (0.7, 1.5)
*HCW characteristics*				
Sex				
Female	1.2 (0.8, 1.9)	0.9 (0.4, 1.8)	0.9 (0.5, 1.7)	1.0 (0.7, 1.4)
Male	1.0	1.0	1.0	1.0
Age (years)				
< 30	1.0	1.0	1.0	1.0
30–39	0.7 (0.4, 1.3)	0.5 (0.2, 1.2)	1.0 (0.6, 1.9)	1.0 (0.6, 1.4)
40–49	1.0 (0.5, 1.7)	0.4 (0.1, 1.0)	0.9 (0.5, 1.5)	0.9 (0.6, 1.4)
50–59	1.3 (0.6, 2.7)	0.5 (0.2, 1.2)	1.0 (0.6, 1.8)	1.2 (0.8, 1.7)
Occupational category				
Medical doctor	0.3 (0.1, 0.7)	NA	NA	NA
Clinical officer	NA	NA	NA	1.1 (0.6, 2.3)
Nurse	0.4 (0.1, 1.0)	0.9 (0.3, 2.7)	1.2 (0.8, 1.9)	1.4 (0.9, 2.0)
Midwife	0.5 (0.2, 1.8)	0.9 (0.3, 3.3)	NA	NA
Medical auxiliary staff	0.3 (0.1, 0.8)	0.9 (0.3, 2.9)	1.2 (0.7, 2.1)	0.8 (0.4, 1.5)
Lab technician or assistant	NA	NA	1.3 (0.7, 2.7)	0.0 (0.0, 0.0)
Community health worker	0.0 (0.0, 0.0)	0.8 (0.3, 2.5)	0.7 (0.4, 1.3)	1.6 (0.4, 6.3)
Non-medical staff^a^	1.0	1.0		1.0
Highest educational level achieved				
Primary school or below	1.0	1.0		1.0
Secondary school	0.9 (0.4, 2.3)	1.3 (0.7, 2.3)	NA	0.6 (0.1, 2.7)
University	0.6 (0.3, 1.1)	0.9 (0.5, 1.8)	1.4 (0.7, 2.6)	0.7 (0.2, 2.9)
Experience (years)				
0–1	1.0	1.0	1.0	1.0
2–4	0.8 (0.3, 2.2)	0.7 (0.3, 1.5)	1.2 (0.7, 2.1)	0.8 (0.4, 1.6)
5–9	0.7 (0.2, 1.9)	0.3 (0.1, 0.7)	0.7 (0.4, 1.2)	0.9 (0.5, 1.7)
10+	0.9 (0.3, 2.3)	0.4 (0.2, 0.8)	1.5 (0.9, 2.4)	1.0 (0.6, 1.6)
Amount worked per week (hours)				
0–19	1.0		1.0	1.0
20–39	3.4 (1.2, 9.9)	NA	0.3 (0.1, 1.0)	1.0 (0.7, 1.5)
40–49	3.6 (1.5, 9.1)	0.3 (0.1, 1.3)	0.4 (0.1, 1.2)	1.0 (0.8, 1.4)
50+	2.7 (1.2, 6.3)	0.4 (0.2, 1.0)	0.4 (0.1, 1.2)	1.0 (0.7, 1.5)
*Knowledge and proficiency*				
Knowledge index				
Lowest	1.0	1.0	1.0	1.0
Highest	0.9 (0.6, 1.5)	0.5 (0.2, 1.0)	0.7 (0.4, 1.1)	1.1 (0.8, 1.5)
RDT proficiency (tercile)				
Lowest	1.0	1.0	1.0	1.0
Middle	0.6 (0.3, 1.1)	1.0 (0.4, 2.9)	2.2 (1.2, 4.0)	1.2 (0.8, 1.8)
Highest	0.7 (0.4, 1.1)	0.5 (0.2, 1.2)	1.4 (0.8, 2.6)	1.1 (0.8, 1.7)
*Attitudes*				
Is it possible for a patient to have a negative RDT test when they actually have a malaria infection?				
Yes	1.1 (0.7, 1.7)	1.2 (0.6, 2.5)	0.7 (0.4, 1.3)	0.9 (0.6, 1.3)
No	1.0	1.0	1.0	1.0
Do you think you should treat a patient with an antimalarial even if their RDT returns a negative result?				
Yes	2.1 (0.9, 4.6)	1.8 (0.7, 4.9)	1.2 (0.6, 2.4)	1.1 (0.8, 1.5)
No	1.0	1.0	1.0	1.0
I have enough time to use malaria RDTs correctly in this facility for all patients who need them				
Agree or strongly agree	0.7 (0.2, 2.3)	0.3 (0.1, 1.0)	0.7 (0.5, 1.0)	1.2 (0.7, 1.9)
Neutral	0.4 (0.1, 1.5)	0.1 (0.0, 0.8)	0.8 (0.2, 3.8)	1.2 (0.7, 2.0)
Disagree or strongly disagree	1.0	1.0	1.0	1.0
If an RDT is negative, I have medicines at this facility that I can use to treat the patient other than antimalarials				
Agree or strongly agree	0.8 (0.5, 1.1)	3.2 (0.8, 12.8)	1.3 (0.7, 2.6)	1.4 (0.8, 2.3)
Neutral	1.1 (0.4, 2.8)	1.3 (0.3, 5.2)	4.4 (0.8, 23.1)	2.1 (1.2, 3.6)
Disagree or strongly disagree	1.0	1.0	1.0	1.0
*Subjective norms*				
I use malaria RDTs because the patients at this health facility expect me to use them				
Agree or strongly agree	0.9 (0.5, 1.5)	1.9 (0.9, 3.7)	1.0 (0.6, 1.8)	1.1 (0.8, 1.5)
Neutral	1.1 (0.5, 2.5)	0.5 (0.2, 1.3)	2.7 (1.6, 4.5)	0.9 (0.6, 1.5)
Disagree or strongly disagree	1.0	1.0	1.0	1.0
I use malaria RDTs because my supervisor expects me to use them				
Agree or strongly agree	0.8 (0.5, 1.3)	1.5 (0.7, 3.0)	1.3 (0.8, 2.3)	1.3 (0.9, 1.8)
Neutral	0.6 (0.3, 1.1)	0.5 (0.3, 0.9)	1.3 (0.6, 3.1)	1.3 (0.7, 2.6)
Disagree or strongly disagree	1.0	1.0	1.0	1.0
I use malaria RDTs because the national malaria treatment guidelines require me to use them				
Agree or strongly agree	0.4 (0.2, 0.9)	1.0 (0.5, 2.1)	0.6 (0.4, 0.9)	0.9 (0.6, 1.3)
Neutral	0.4 (0.2, 0.8)	0.8 (0.4, 1.5)	NA	0.7 (0.4, 1.2)
Disagree or strongly disagree	1.0	1.0	1.0	1.0
*Behaviours*				
Frequency of recording RDT results				
Very often (every day)	1.1 (0.6, 1.8)	0.7 (0.3, 1.4)	2.0 (1.2, 3.3)	1.6 (1.1, 2.3)
Once in a while to often	0.7 (0.2, 3.5)	0.5 (0.3, 0.9)	3.1 (1.2, 7.5)	1.7 (1.2, 2.5)
Never	1.0	1.0	1.0	1.0
*Training and supervision*				
Received RDT training in the past year				
Yes	0.9 (0.5, 1.4)	0.9 (0.4, 1.9)	0.6 (0.4, 1.0)	1.0 (0.7, 1.4)
No	1.0	1.0	1.0	1.0
Supervisor observed the performance of an RDT within the past year				
Yes	1.4 (0.9, 2.3)	0.6 (0.3, 1.1)	1.2 (0.7, 2.0)	1.1 (0.8, 1.6)
No	1.0	1.0	1.0	1.0
*RDT-level variables*				
Patient sex				
Female	1.1 (0.8, 1.6)	1.1 (0.9, 1.3)	1.3 (1.1, 1.6)	1.3 (1.1, 1.5)
Male	1.0	1.0	1.0	1.0
Patient age (years)				
< 5	1.0	1.0	1.0	1.0
5–14	0.5 (0.3, 0.7)	1.0 (0.7, 1.4)	0.6 (0.5, 0.8)	1.0 (0.8, 1.3)
15+	2.9 (2.0, 4.2)	3.0 (2.2, 4.0)	1.3 (1.0, 1.6)	3.0 (2.3, 3.9)

^a^The non-medical staff category includes students, interns and volunteers

#### Multivariable

**Fig. 2:**
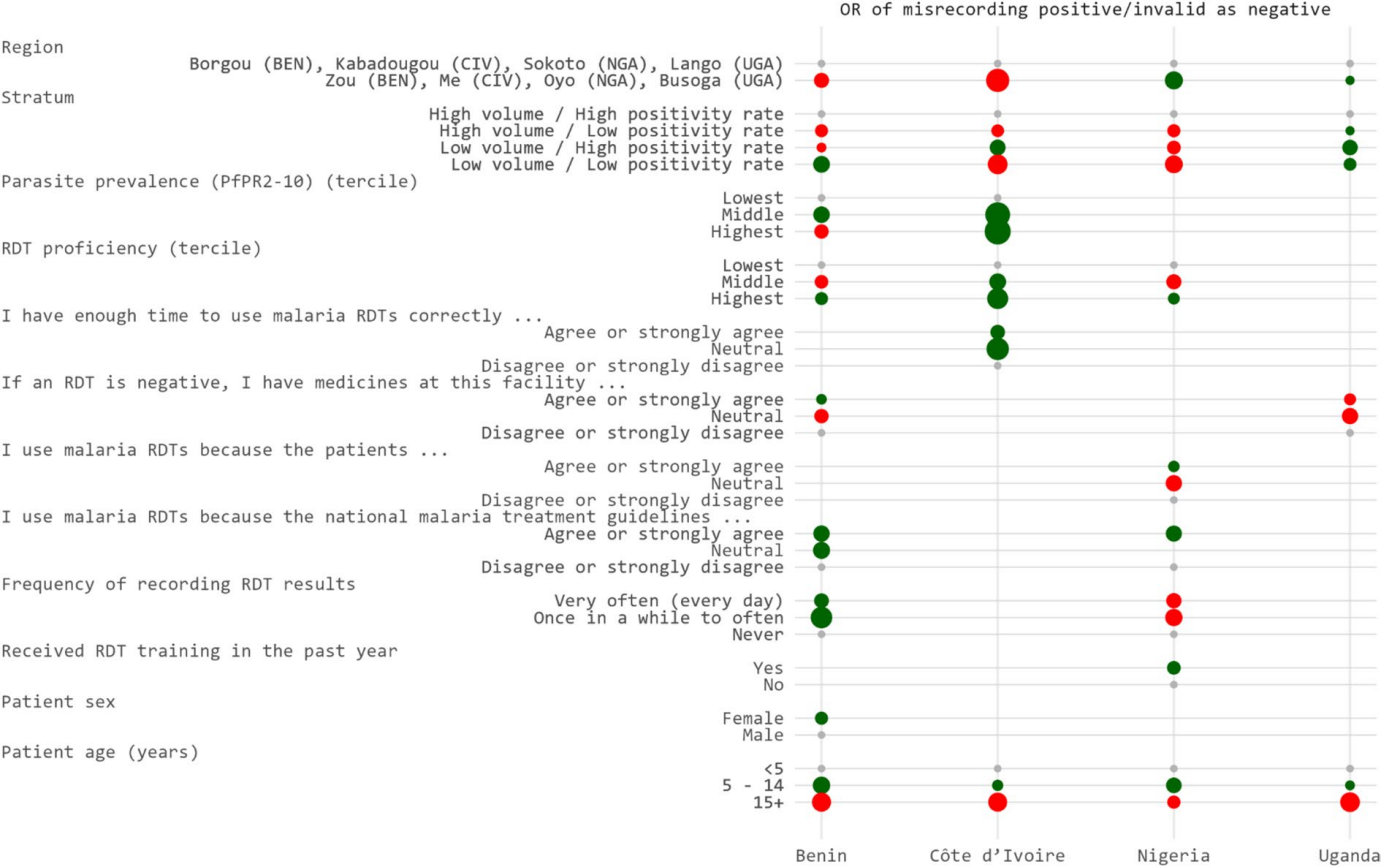
Dot plot of adjusted odds ratios for factors selected in final models of predictors of misrecording RDT results as negative by country, 2023

In the multivariable models assessing misrecording of positive or invalid RDT results as negative, no HCW characteristics were retained in the final model for any country (Fig. 2). PfPR_2–10_ was retained for Benin and Côte d’Ivoire, but in the latter country, higher parasite prevalence was protective against misrecording results as negative while in Benin, results were inconsistent. Patient age remained the most consistent predictor across countries: school-aged children (5–14 years) were less likely to have their results misrecorded as negative compared to children < 5, while patients aged 15 years and older were more likely to be affected by this type of misrecording.

## Discussion

The initial report from this multi-country evaluation documented that between 5.1 and 7.3% of malaria RDT results were misrecorded as positive, and between 0.7 and 3.7% were misrecorded as negative, across 64 health facilities in Benin, Côte d'Ivoire, Nigeria, and Uganda [17]. Building on those findings, this analysis, based on over 100,000 RDTs performed by 499 HCWs, examined which characteristics of health facilities, HCWs, and patients for whom RDTs were performed were associated with inaccurate recording of malaria RDT results. Our results indicate that the factors associated with misrecording RDT results differed depending on whether the original test result was positive or negative. Broadly, HCW characteristics were more likely to be associated with misrecording negative or invalid results as positive than with misrecording positives or invalids as negative, suggesting that the former may be more influenced by individual decision-making. This finding aligns with previous multi-country studies showing HCWs in endemic areas often override negative RDT results based on clinical suspicion [13]. Although few factors were consistent across all four countries, these findings suggest new directions for future evaluations aimed at understanding HCW motivations and identifying contextual factors that promote accurate recording of RDT results.

Malaria prevalence and facility-level factors emerged as important predictors of misrecording. The inclusion of region and stratum in final models ensured adjustment for sampling design but results also suggest that contextual differences within countries influence recording behaviour. The estimated PfPR_2-10_ surrounding the facility was retained for three of the four country models for misrecording results as positive. In Côte d'Ivoire, Nigeria, and Uganda, the highest level of parasite prevalence was associated with increased odds of misrecording a negative result as positive. This pattern may reflect a tendency among HCWs in higher transmission settings to expect a greater proportion of suspected cases to have malaria and to question the validity of negative results, potentially leading them to prescribe antimalarials and record the RDT result as positive to align with the treatment decision [13]. Similar findings were reported in Kenya, where HCWs in high-transmission areas were 2.3 times more likely to disregard negative RDT results [7].

Educational attainment was also retained in three of the four countries (Côte d'Ivoire, Nigeria, and Uganda), with those holding university degrees or those completing secondary school significantly less likely to misrecord results than those with primary-level education or less. This supports findings from a study showing that advanced education improves adherence to test results when combined with regular supervision [22]. However, previous studies suggest that more highly trained HCWs may feel confident overriding diagnostic test results based on clinical judgment than lower cadre staff [23].

Notably, none of the variables intended to capture HCWs' specific knowledge or skills related to RDTs, such as observed RDT proficiency, recent training, or supervisory oversight, were consistently associated with improved recording accuracy. This finding seems somewhat counterintuitive, as one could expect that better technical skills, regular training, or close supervision would directly translate into more accurate documentation. However, the absence of such associations highlights that recording accuracy is likely influenced by factors beyond technical competency. This can be further explained in three ways. First, recording practices are often shaped by systemic and contextual factors such as workload, staffing shortages, documentation burden, and competing clinical priorities. Even if HCWs are proficient at performing RDTs, they may record results inaccurately due to time pressure, lack of incentives, or weak accountability structures. Second, the persistence of inaccurate recording despite training suggests that knowledge alone is insufficient to change behaviour; primordial habits, workplace culture, or a lack of functional monitoring and evaluation systems may undermine the impact of prior capacity-building initiatives. Third, the limited effect of supervisory oversight indicates that supervision may be too infrequent, superficial, or not sufficiently focused on reinforcing accurate recordkeeping.

Higher educational attainment may reflect a broader understanding of diagnostic principles, increased confidence in test results, or a greater capacity to resist external pressures, such as patient expectations, that could influence decision-making and documentation.

While this multi-country analysis identifies some commonalities, it is important to highlight key differences between the countries that may have shaped HCWs’ recording practices. For example, Benin showed the highest overall agreement with the external panel (94.3%) and the lowest proportion of results misrecorded as negative, which may in part be attributed to ongoing RDT quality assurance programmes and validation studies conducted in the country. In contrast, Nigeria had relatively lower agreement (90.5%) but a higher proportion of HCWs reporting recent RDT training and supervisory oversight, suggesting that while these supports were more available, other contextual pressures like high patient volumes or greater reliance on non-clinical staff may have affected accuracy. Côte d’Ivoire and Uganda demonstrated lower rates of performing RDTs very often compared to Benin and Nigeria, which might reflect differences in health system prioritization of diagnostic confirmation or differences in the workload of frontline staff.

The variations observed across countries may also relate to health system structures, including differences in supervision intensity, the presence of national malaria control interventions, and the local epidemiology of malaria transmission [22]. For instance, higher malaria prevalence in parts of Côte d’Ivoire and Uganda could contribute to greater diagnostic suspicion among HCWs, leading to a higher rate of negative results being misrecorded as positive. Conversely, in Nigeria, where community health workers and laboratory assistants made up a greater proportion of test performers, the observed association between cadre and misrecording suggests that staff with less formal clinical training may require tailored support to improve recording accuracy. Similarly, Benin was unique in having a higher proportion of medical doctors among RDT performers; however, there was no statistically significant difference in misrecording between medical doctors and non-medical staff. These contextual nuances show that a one-size-fits-all approach to improving RDT recording may not be effective, and interventions should consider local health system resources, staff composition, and ongoing malaria programme activities. These country-specific comparisons underscore opportunities to leverage strengths in each setting. For example, the strong supervisory culture reported in Nigeria might be built upon to provide targeted mentorship to cadres with higher misrecording rates, while the RDT validation work in Benin could serve as a model for periodic refresher exercises elsewhere.

Attitudinal and behavioural factors were also influential. In three countries, HCWs who believed patients should be treated with antimalarials even after a negative RDT were more likely to misrecord results as positive. This belief could reflect an understanding of the limit of detection of RDTs that were not designed to pick up very low density infections, or it could reflect concern that recent use of antimalarials had rendered infections undetectable [12]. While it may appear fairly self-evident that HCWs with this belief would also be more likely to misrecord negative results as positive, it is notable that this association persisted even after controlling for other HCW characteristics, facility-level factors, malaria prevalence and patient demographics, suggesting that personal beliefs about treatment practices may independently shape recording behaviour. This underscores the need for behavioural interventions alongside technical training, as demonstrated in a recent cluster-randomized trial in Tanzania [24].

Fewer factors were associated with misrecording a positive or invalid result as negative, and this type of misrecording occurred less frequently across all countries. Nonetheless, regional differences within countries persisted, highlighting the potential influence of contextual and health system factors on HCW recording behaviour. PfPR_2–10_ was retained in two of the four country models and was associated with reduced odds of misrecording positive results as negative, though the strength and direction of this relationship were not consistent. One plausible explanation is that HCWs practicing in areas with higher malaria prevalence may have expected to see more positive cases, thereby reinforcing their trust in a positive test result. Notably, the highest level of RDT proficiency was associated with decreased odds of misrecording results as negative in three countries. This pattern, which was not observed for misrecording results as positive, suggests that a proportion of these errors may stem from incorrect RDT administration or interpretation. For example, less proficient HCWs may have read the RDTs prematurely, before the full development time had elapsed, and incorrectly classified results as negative. Since research assistants photographed the RDTs after the HCW had interpreted them, even a few minutes' delay could have allowed a test line to appear, resulting in a positive interpretation by the panel.

Patient age emerged as a key factor associated with both types of misrecording. Compared to children under five, school-aged children were more likely to have results misrecorded as positive but less likely to have results misrecorded as negative. Patients aged 15 years and older were at increased risk of both types of misrecording in some settings. These patterns may reflect differences in clinical expectations, diagnostic uncertainty, or patient preferences across age groups. The increased misrecording for adults is particularly concerning given the growing recognition of adult malaria burden in endemic areas [25]. To date, age-related differences in adherence to RDT results and accuracy of RDT recording have not been reported. This warrants further investigation to better understand how and why patient age affects treatment decisions and the reliability of surveillance data.

Taken together, these findings suggest that distinct mechanisms may underlie the misrecording of negative versus positive RDT results. Misrecording negative or invalid results as positive appears more strongly influenced by health worker characteristics and beliefs, particularly perceptions of malaria risk and the perceived necessity of treatment. In contrast, misrecording results as negative may reflect broader contextual influences or procedural errors. Both types of misrecording were associated with patient age, which may serve as a proxy for patient expectations or preferences. From a public health perspective, these errors have different implications: false positives lead to overtreatment and drug pressure, while false negatives result in missed cases and continued transmission. These results underscore the multifactorial nature of inaccurate RDT reporting. While targeted interventions could address specific contributing factors, a more pragmatic and potentially more effective strategy may be to motivate health workers to improve recording accuracy through regular validation and feedback on HMIS data.

This study had several strengths, including a large sample size, the use of directly observed RDT images for classification, and harmonized data collection across multiple countries. However, the findings may have limited generalizability, as the study was restricted to public health facilities in purposively selected regions supported by PMI. Furthermore, some potentially relevant variables may not have been measured. Future research should incorporate qualitative methods to explore attitudes and perceptions related specifically to misrecording of results, which could help identify and measure indicators more directly aligned with this behaviour.

## Conclusion

Misrecording of RDT results in health facility registers is a measurable and non-negligible problem in sub-Saharan Africa. The determinants of misrecording RDT results vary, with HCW beliefs and qualifications playing a prominent role in misrecording results as positive and contextual factors and HCW proficiency shaping misrecording results as negative, and patient age modifying both. Improving the reliability of malaria surveillance data based on RDTs may require interventions that directly motivate HCWs to report accurately.

## Supplementary Information

Additional file 1.

## Data Availability

The datasets used and/or analysed during the current study can be provided by the corresponding author on reasonable request.

## Abbreviations

AI: Artificial intelligence
CI: Confidence interval
HCW: Health care worker
HMIS: Health management information system
IQA: Image quality assurance
ODK: Open data kit
PMI: President’s malaria initiative
RA: Research assistant
RDT: Rapid diagnostic test
SOP: Standard operating procedure
TPR: Test positivity rate
