# Supplementary material for: Characteristics of healthcare workers and health facilities associated with inaccurate recording of malaria rapid diagnostic test results: a multi-country study
Source: Malar J. 2025 Nov 27;25:4. doi: 10.1186/s12936-025-05674-2 (PMC12764150; doi:10.1186/s12936-025-05674-2)
Supplement: Supplementary file 1 — Additional file 1. [file 12936_2025_5674_MOESM1_ESM.docx]

Supplemental table 1. Algorithm to create knowledge index

| **Algorithm** | **Score** |
| --- | --- |
| Out of list of false causes of malaria, only the correct answer, ‘mosquito’, is selected | 1 |
| Selected insecticide-treated nets or bednets as a protective measure against malaria | 1 |
| Selected use of antimalarial medicine as a protective measure against malaria | 1 |
| Selected any of the vector control methods (protection against mosquito bites, use of screens, use of repellent, indoor residual spraying) as protective measures against malaria | 1 |
| Choose fever as a sign of malaria and none of the incorrect signs | 1 |
| Chose ‘high fever’ as a sign of severe malaria and none of the incorrect signs | 1 |
| Total | 6 |

**Supplemental table 2. Explanation for inclusion or exclusion of variables from the final model selection.**

| **Variable** | **OR of misrecording true negative as positive** | | **OR of misrecording true positive as negative** | |
| --- | --- | --- | --- | --- |
|  | **Included in stepwise** | **Reason** | **Included in stepwise** | **Reason** |
| Region | Yes | Potential for confounding | Yes | Potential for confounding |
| Stratum | Yes | Reflects sampling strategy | Yes | Reflects sampling strategy |
| Parasite prevalence (PfPR2-10) (tercile) | Yes | Statistical association in at least 1 country | Yes | Statistical association in at least 1 country |
| Sex | Yes | Statistical association in at least 1 country | No | No statistical association |
| Age (years) | Yes | Statistical association in at least 1 country | No | No statistical association |
| Occupational category | No | To much variability by country in categories | No | To much variability by country in categories |
| Highest educational level achieved | Yes | Statistical association in at least 1 country | No | No statistical association |
| Experience (years) | Yes | Statistical association in at least 1 country | No | No statistical association |
| Amount worked per week (hours) | Yes | Statistical association in at least 1 country | No | No statistical association |
| Knowledge index | Yes | Statistical association in at least 1 country | No | No statistical association |
| RDT proficiency (tercile) | No | No statistical association | Yes | Statistical association in at least 1 country |
| Is it possible for a patient to have a negative RDT test when they actually have a malaria infection? | Yes | Statistical association in at least 1 country | No | No statistical association |
| Do you think you should treat a patient with an antimalarial even if their RDT returns a negative result? | Yes | Statistical association in at least 1 country | No | No statistical association |
| I have enough time to use malaria RDTs correctly in this facility for all patients who need them | Yes | Statistical association in at least 1 country | Yes | Statistical association in at least 1 country |
| If an RDT is negative I have medicines at this facility that I can use to treat the patient other than antimalarials | Yes | Statistical association in at least 1 country | Yes | Statistical association in at least 1 country |
| I use malaria RDTs because the patients at this health facility expect me to use them | No | No statistical association | Yes | Statistical association in at least 1 country |
| I use malaria RDTs because my supervisor expects me to use them | Yes | Statistical association in at least 1 country | No | No statistical association |
| I use malaria RDTs because the national malaria treatment guidelines require me to use them | Yes | Statistical association in at least 1 country | Yes | Statistical association in at least 1 country |
| Frequency of performing RDTs | No | No statistical association | No | Colinear with frequency of recording RDT results |
| Frequency of recording RDT results | Yes | Statistical association in at least 1 country | Yes | Statistical association in at least 1 country |
| Received RDT training in the past year | No | No statistical association | Yes | Statistical association in at least 1 country |
| Supervisor observed their performance of an RDT in the past year | Yes | Statistical association in at least 1 country | No | No statistical association |
| Patient sex | Yes | Statistical association in at least 1 country | Yes | Statistical association in at least 1 country |
| Patient age (years) | Yes | Statistical association in at least 1 country | Yes | Statistical association in >1 country |
